# Implementation outcomes of a culturally adapted diabetes self-management education intervention for Native Hawaiians and Pacific islanders

**DOI:** 10.1186/s12889-020-09690-6

**Published:** 2020-10-20

**Authors:** Kaʻimi A. Sinclair, Anna Zamora-Kapoor, Claire Townsend-Ing, Pearl A. McElfish, Joseph Keaweʻaimoku Kaholokula

**Affiliations:** 1 Institute for Research and Education to Advance Community Health, College of Nursing, Washington State University, 1100 Olive Way, Suite 1200, Seattle, WA 98101 USA; 2 Institute for Research and Education to Advance Community Health, Washington State University, 1100 Olive Way, Suite 1200, Seattle, WA 98101 USA; 3 Department of Native Hawaiian Health, John A. Burns School of Medicine, University of Hawaiʻi, 677 Ala Moana Blvd, Suite 1016, Honolulu, Hawaiʻi 96813 USA; 4 University of Arkansas Medical Sciences, 1125 N. College Avenue, Fayetteville, AR 72703-1908 USA

**Keywords:** Diabetes self-management education, Native Hawaiian, Pacific Islander, Culturally adapted, Implementation research, Implementation outcomes, Community-engaged research, Peer educator

## Abstract

**Background:**

Native Hawaiians and Pacific Islanders (NHPIs) experience a disproportionate burden of type 2 diabetes and related complications. Although diabetes self-management education and support (DSMES) interventions have generally yielded positive results, few NHPIs have been included in these studies, and even fewer studies have been evaluated using a randomized controlled trial design and/or implementation research methods. The purpose of this pilot study was to evaluate implementation outcomes of a culturally adapted diabetes self-management education intervention delivered by peer educators to Native Hawaiians and Pacific Islanders residing in Honolulu, Hawai’i.

**Methods:**

In three study sites, the peer educators and 48 participants randomized to the intervention were invited to participate in the mixed methods implementation research. We used a convergent parallel design to collect implementation data including fidelity, feasibility, acceptability, appropriateness, adoption, and sustainability. Data were collected from class observations, participants’ class feedback, and post-intervention focus groups with participants and peer educators.

**Results:**

In 314 end-of-class feedback surveys, 97% of respondents expressed that they were satisfied or highly satisfied with the class content and activities, 98% reported that the classes and materials were very useful, 94% reported very applicable, and 93% reported materials were culturally appropriate. Respondents identified several aspects of the program as especially enjoyable: interactions with peer educators, meeting in groups, learning about other participants’ experiences with diabetes, and the information presented in each class. Major themes that emerged from the end-of-intervention focus groups were the relevance of the educational materials, strategies to manage blood glucose, hands-on activities, cultural aspects of the program, including the stories and analogies used to convey information, and appreciation of the group format and peer educators.

**Conclusions:**

Results from this research support a culturally tailored, peer educator approach to DSMES among NHPIs. Delivery of the Partners in Care program is feasible in health care and community settings and is a reimbursable DSMES program.

**Trial registration:**

Clinicaltrials.gov Identifier: NCT01093924 prospectively registered 01.20.09.

**Supplementary information:**

The online version contains supplementary material available at 10.1186/s12889-020-09690-6.

## Background

Native Hawaiians and Pacific Islanders (NHPIs) experience a disproportionate burden of type 2 diabetes and related complications [1–5]. Among NHPIs in Hawaii, the prevalence of type 2 diabetes ranges from 19 to 22%, [3] and it is the fourth-leading cause of death [3]. The extent of unmanaged diabetes among NHPIs is evident in the large number of reported medical complications and preventable hospitalizations related to type 2 diabetes [3, 5, 6].

Type 2 diabetes has been recognized as one of the most challenging chronic illnesses to manage [7, 8]. Early detection, treatment, and appropriate self-management (e.g., healthy eating, regular exercise, and medication use) are critical to reducing the risk of complications and improving quality of life [9]. Two landmark clinical trials have demonstrated that tight control of blood glucose can significantly reduce the risk of diabetes complications [10, 11]. Every 1% reduction in A1C (a measure of glycemic control using levels of glycosylated hemoglobin) leads to a 14% reduction in the risk of all-cause mortality and myocardial infarction, and a 37% reduction in the risk of microvascular complications.

Interventions that promote diabetes self-management education and support (DSMES) have proven effective at improving glycemic control and encouraging lifestyle behaviors that can reduce the risk of diabetes complications and improve quality of life. DSMES aims to support informed decision-making, self-care behaviors, problem solving, and active collaboration between patients and their healthcare team [12]. The clinical goal for A1C, an average of blood glucose over 3 months, is < 7% to prevent microvascular complications [9]. NHPIs are more likely than members of other populations to have A1C > 8%, [5] have high energy intake, be physically inactive, and be overweight or obese [13–18]. Given the disproportionate burden of diabetes [3] and challenges in managing diabetes [5], identifying appropriate DSMES interventions for NHPIs is a public health priority. Although DSMES interventions have generally yielded positive results, few NHPIs have been included in these studies, and even fewer studies have been evaluated using a randomized controlled trial design and/or implementation research methods [3]. Therefore, in 2011, Partners in Care was implemented in Honolulu, Hawai’i as a randomized controlled trial to evaluate the effectiveness of a DSMES intervention on glycemic control and self-management knowledge and behaviors among NHPIs.

This paper describes the evaluation of the Partners in Care implementation to understand the process of delivering a culturally adapted DSMES intervention delivered by trained peer educators in low-resource community and clinic settings. Implementation research is used to evaluate strategies that may optimally increase adoption and implementation of evidence-based practices and treatments in real-world settings [19]. It is a multidisciplinary method that evaluates implementation from the perspective of the users of research [20]. Users may include healthcare systems or providers who want to identify how to best integrate an evidence-based treatment into their particular context; organizations and communities who want to understand contextual adaptations that will improve the implementation of evidence-based practices; and participants in health promotion and disease prevention research [19]. Understanding facilitators and challenges to program implementation can inform the transferability, scalability, and dissemination of effective programs to other populations and settings [21, 22]. Proctor and colleagues [23] describe a taxonomy of 8 conceptually distinct implementation outcomes – acceptability, adoption, appropriateness, feasibility, fidelity, implementation cost, penetration, and sustainability. Our study evaluated, and this paper presents the results of, acceptability, adoption, appropriateness, feasibility, fidelity, and sustainability. These findings may be used to inform future implementation of Partners in Care with other NHPIs and in other populations and settings.

## Methods

### Partners in Care Intervention Development and Delivery

The Partners in Care intervention was adapted from previously evaluated DSMES interventions conducted with American Indians in the Southwest [24] and with African Americans and Latinos in Detroit, Michigan [25]. Adaptation procedures have been detailed elsewhere [26–28]. Briefly, 4 focus groups with 38 NHPI community members to assess facilitators and challenges to self-management of diabetes and interest in a group-based educational intervention informed adaptations. Major focus group themes included multi-level difficulties in adherence to self-management behaviors, such as an eating plan recommended for glucose control, increased physical activity to lose or maintain a healthy weight, and lack of knowledge about the importance of glucose control to prevent complications. These themes were similar to those found among other populations the authors have worked with, [29] and strategies to address several challenges were already included in the curriculum. The focus groups themes and feedback from a community-academic steering committee [30] and 3 NHPI peer educators were combined with evidence-based American Diabetes Association clinical guidelines for blood glucose, A1C, blood pressure, and lipids [31] and Social Cognitive Theory [32] to inform the 12-lesson Partners in Care curriculum (Table 1).

**Table 1:** Partners in Care 12 weekly 1-h curriculum topics and content

Topic	Content
Lesson 1: Glucose Balance Makes a Difference	Description and illustrations of what blood glucose and A1C numbers mean; recommended goals for blood glucose and A1C; how to lower blood glucose.
Lesson 2: Medicine and Glucose Balance	Description and brochures for each diabetes medication; possible side effects; brief description of how each medicine works in the body to lower blood glucose; how to treat hypoglycemia; why it is important to take medication as prescribed and to tell provider about side effects.
Lesson 3: Food and Glucose Balance	How various types of foods can affect blood glucose; strategies to keep blood glucose balanced throughout the day.
Lesson 4: Plan Meals for Glucose Balance	How to plan healthy meals for blood glucose balance; how to count carbohydrates and read labels.
Lesson 5: Move More, Sit Less!	Increase physical activity through discussion of challenges to exercise; skills training through role playing, and behavior therapy using goal setting, reinforcement, modeling, and identification of non-food rewards.
Lesson 6: Diabetes and a Healthy Heart	Description of how diabetes affects the heart; how to control high blood pressure; recommended goals for blood pressure.
Lesson 7: Diabetes and Cholesterol	Description of total cholesterol, HDL, LDL, and triglycerides; foods that contain which types of cholesterol; how to control high cholesterol; recommended goals for lipids.
Lesson 8: Healthy Feet Keep You Going!	Importance of checking one’s feet every day; how to check one’s feet; getting feet checked by provider; how to identify appropriate shoes to avoid injury to feet; what to do if you have a sore that won’t heal.
Lesson 9: Stress, Depression, and Diabetes	Description of the relationship between diabetes, stress, and depression; how to lower stress; how to know if one is depressed; what to do for depression; 3 stress-reducing strategies.
Lesson 10: Preventing Complications	Discussion of other diabetes-related complications (dental, neuropathy, nephropathy, retinopathy) and how to prevent or delay them.
Lesson 11: Your Diabetes Team	How to better communicate with one’s healthcare team; whom to include in one’s diabetes team; the roles of each person; role playing to practice how to ask questions of providers.
Lesson 12: Living Well with Diabetes	Maintaining self-management activities in order to stay healthy with diabetes and prevent or delay complications; potluck meal of healthy foods during the final lesson.

The authors provided a 4-h study protocol and curriculum implementation training to the three NHPI peer educators and provided technical assistance throughout the study. One peer educator worked for a Native Hawaiian community organization as a community-health worker, and 2 peer educators worked in Federally Qualified Health Centers, one as a health educator and the other as a registered dietician. The peer educators were well positioned to deliver Partners in Care because they had just completed delivery of a weight loss education intervention in their communities and received prior training in group facilitation, intervention research, behavior change strategies, survey administration, and recruitment and retention methods.

Participants received a $20 gift card for each data collection visit, a copy of the curriculum, and incentives during each class that were relevant to the topic of the class. For example, a pill box was offered during Lesson 2: Medicine and Glucose Balance; a stretch band was offered during Lesson 5: Move More, Sit Less!; and a colander to rinse and drain fat from cooked ground beef was provided in Lesson 7: Diabetes and Cholesterol. Informational brochures and recipes from the American Diabetes Association were also included in the curriculum as supplemental material. The study protocol was approved by the University of Hawai’i Committee on Human Studies and the Native Hawaiian Health System Institutional Review Board.

### Partners in Care Intervention Outcomes

Clinical and behavioral outcomes from the Partners in Care intervention have been described in detail elsewhere [28]. Briefly, a total of 82 NHPI were recruited, gave written informed consent, and completed the baseline assessment that was developed for this study (Supplementary File 1). Participants were randomly assigned to either the intervention or a wait list control group who received the same intervention after the final follow up survey. At 3 months, there were significant baseline adjusted differences between the 48 participants randomized to the Partners in Care intervention compared to the and 34 wait list control group participants in intent-to-treat (*p* < 0.001) and complete case analyses (*p* < 0.0001) for A1C, understanding (p < 0.0001), and performing diabetes self-management (p < 0.0001) [28]. The Partners in Care study adheres to CONSORT guidelines [28].

### Implementation research: study design, setting, and participants

Three peer educators and 48 participants in the three study sites who were randomized to the Partners in Care intervention were invited to participate in the implementation research. A convergent parallel design was used throughout the intervention to collect implementation data to evaluate acceptability, adoption, appropriateness, feasibility, fidelity, and sustainability (Table 2). With a convergent parallel design, both qualitative and quantitative data are collected, each is analyzed separately, then the data are merged and interpreted together.

**Table 2:** Measurement of implementation outcomes for Partners in Care participants and peer educators

Implementation Outcome	Definition	Partners in Care Data Source	Partners in Care Timepoint
Participants			
Acceptability	Satisfaction, usefulness, applicability, and cultural appropriateness of intervention materials and content	4-item ranked questions	End of each of 12 classes
		3 open-ended questions	End of each of 12 classes
		Attendance	Beginning of each of 12 classes
		Focus groups	End of final class in each site
Adoption	Intention, initial decision, or action to try self-management behaviors	3 open-ended questions	End of each of 12 classes
		Follow-up data collection	3-months post-baseline data collection
Appropriateness	Perceived fit, relevance, or compatibility of the evidence- based practice for a given consumer	Participation and retention	Beginning of each of 12 classes
		Focus groups	End of final class in each site
Peer Educators			
Acceptability	Satisfaction with aspects of the intervention (e.g., content, complexity, comfort, delivery, and credibility)	Focus group	End of the final class
Adoption	Intention, initial decision, or action to try an evidence-based practice; initial implementation	Delivery of intervention	Throughout intervention
Appropriateness	Perceived fit, relevance, or compatibility of evidence-based practice in a given setting and/or perceived fit of the program to address a particular issue or problem	Focus group	End of final class
Feasibility	Extent to which a new treatment or innovation can be successfully used or carried out within a given setting; enabling factors and challenges to implementation	Focus group	End of final class
Fidelity	Degree to which an intervention was implemented as it was prescribed in the original protocol	Direct observation of peer educators by research staff	Throughout intervention
Organization/Community			
Sustainability	Extent to which a newly implemented treatment is maintained or institutionalized within a community or service setting’s ongoing, stable operations	Peer educators, communication with adopters	Upon study completion

### Participant implementation measures

To measure class attendance, participants signed in at the beginning of each class and these data were entered into a participant attendance database. To measure overall acceptability of each class, defined as satisfaction, usefulness, applicability, and cultural appropriateness, participants were asked to respond to 4 items at the conclusion of every class. General satisfaction with each class was rated as 1 = highly unsatisfied; 2 = somewhat unsatisfied; 3 = neither unsatisfied nor satisfied; 4 = somewhat satisfied; or 5 = highly satisfied. To rate the usefulness of each class and its materials, the applicability of information, and the cultural appropriateness of class materials, participants used the rating scale 1 = not at all; 2 = somewhat; or 3 = very for each question. Participants were also asked to respond to 3 open-ended questions: 1) What information during the class did you find especially helpful?; 2) What goal did you set for this class?; and 3) Provide any other comments you would like to share about today’s meeting. After the final class, research staff conducted a 1-h focus group with each group of intervention participants to assess their satisfaction with the intervention, the usefulness and applicability of the information provided, and the perceived impact of the intervention on diabetes self-management knowledge and behaviors. To discourage socially desirable answers, peer educators were not present during these focus groups.

### Peer educator implementation measures

To measure fidelity to the study protocol, two research staff concurrently but independently observed 4 random classes of each peer educator (*n* = 12 classes). Each research staff member was trained by the principal investigator to complete a checklist of critical intervention behaviors and materials to be delivered during each class to monitor: 1) fidelity to the curriculum, 2) participants’ questions, 3) information added by peer educators, and 4) research staff’s overall impressions of participant understanding and satisfaction with content and activities. During each class, research staff followed along in the curriculum to assess fidelity to the curriculum content. Fidelity was rated on a 1 to 5 scale, with 1 = did not follow curriculum at all (0% fidelity); 2 = followed very little of curriculum (up to 30% fidelity); 3 = followed at least half of the curriculum (50% fidelity); 4 = followed most of the curriculum (up to 80% fidelity); 5 = followed all of curriculum (100% fidelity). While peer educators were trained to adhere to the study protocol and deliver the curriculum as written, they were provided flexibility to incorporate relevant analogies and examples to appropriately convey the information to their groups. Additional information and/or strategies implemented during the study were documented by peer educators and research staff. Research staff’s overall impressions of participant understanding of content and activities was ranked as 1 = no one understood; 2 = very few understood; 3 = some understood; 4 = most understood; 5 = all understood. Overall impressions of participant satisfaction with content and activities was ranked as 1 = not satisfied at all; 2 = somewhat satisfied; 3 = mostly satisfied; 4 = very satisfied.

After the intervention concluded, all 3 community peer educators participated in a focus group that solicited their perceptions of the cultural relevance, acceptability of the intervention materials, appropriateness and feasibility of Partners in Care to improve diabetes self-management in NHPIs, and factors that enabled or challenged intervention implementation. The focus group was conducted as an open, informal discussion, with the academic principal investigator audio-recording peer educators’ remarks.

### Data analysis

All focus groups were audio-recorded, transcribed verbatim, and analyzed using Atlas ti 5.0 (Scientific Software Development, Berlin, Germany, 1998). Miles and Huberman’s [33] method of content analysis, which involves categorizing statements and marking them to reflect concepts or themes, was applied qualitative data. Two coders reviewed the data separately to develop thematic categories based on the topics of the focus group guide. Data reduction was accomplished by selecting, simplifying, abstracting, and coding data according to the preliminary schema. Matrices were developed along thematic lines, and common themes were determined by grouping responses by the question that elicited them. For the end-of-class 3-item open-ended feedback surveys, responses and free-form notes were coded and enumerated, and the number of feedback forms with at least 1 written comment was reported. Descriptive statistics were used to examine participant characteristics, frequency of class participation, participant responses to the 4-item end of class survey to evaluate satisfaction, usefulness, applicability, and cultural appropriateness of class materials, and leading reasons for non-participation in classes. All data were then evaluated together to identify convergence and/or divergence between qualitative and quantitative data. All quantitative analyses were performed using SPSS version 20.0 (SPSS, Inc., Chicago, IL).

## Results

### Participant implementation outcomes

Demographic characteristics have been presented elsewhere [28]. As reported in our outcomes paper, of 82 enrolled participants, 48 were randomized to the Partners in Care intervention and 34 to the wait-list control group. Community partners requested randomizing more participants to the immediate intervention, rather than to the wait list control group. The average age was 53 years in the intervention group and 55 years in the control group. More than half of participants in each group were female and married, but intervention participants were more likely than controls to have a high school education or less. Control participants also had higher systolic blood pressure than those in the intervention group, but blood pressure was high in both groups.

### Class attendance and attrition

Ten participants randomized to the intervention group dropped out before the study began because they could not attend scheduled classes due to competing demands of work and family. Four participants randomized to the intervention and three randomized to the wait list control group could not be reached to determine their reasons for dropping out of the study. Dropout was significantly associated with younger age (*p* = 0.001), but did not vary by site, sex, educational status, or baseline A1C [28]. The 34 intervention participants who remained in the study attended at least 1 class, 70% attended at least 9 classes, and 58% attended all 12 classes. Class size ranged from 6 to 15 participants per site.

### Acceptability and adoption

Intervention participants submitted 314 end-of-class feedback surveys. Survey response rates ranged from 74 to 95% across all 12 classes; 97% of respondents expressed that they were satisfied or highly satisfied with the class content and activities, 98% reported that the classes and materials were very useful, 94% reported very applicable, and 93% reported materials were culturally appropriate. Respondents identified several aspects of the program as especially enjoyable: interactions with peer educators, meeting in groups, learning about other participants’ experiences with diabetes, and the information presented in each class. For example, one participant stated, “Every time I come to class, I learn more about myself and what I need to do to control my blood glucose.” Another wrote, “Now I can teach my daughter how to help me. She always asks how she can help.” In response to the question, “What information did you find especially useful?” 98% of respondents cited class content, including taste testing, label reading, role playing, medication brochures, and problem-solving activities (Table 3). Ninety-seven percent of participants who completed the feedback forms reported goals they set for themselves that were related to class topics.

**Table 3:** Participant responses regarding the Partners in Care class content found most useful (*n* = 314)

Response Topic	Participant Responses
Diabetes self-management class content	•The role of blood glucose, insulin, and the pancreas in diabetes
	•How self-monitoring can show blood glucose level at any time and can help prevent the consequences of very high or very low blood glucose
	•The benefits of diabetes medications, how they control blood glucose, and side effects
	•What blood glucose and A1C numbers mean and what they should be to prevent complications
	•What my blood pressure numbers mean
	•Good cholesterol versus bad cholesterol
	•How to ask family and friends for help with self-management
	•Participating in group discussions to share experiences and ideas
Diet and physical activity	•Increased awareness and understanding of relationship between food and blood glucose
	•How to read food labels to reduce dietary fat and sugar
	•Importance of getting family included in healthy eating and exercise to prevent diabetes
	•Alternative cooking methods such as grilling, steaming, baking
	•Healthier serving sizes of foods
	•The role of physical activity and how it can improve blood glucose, blood pressure, and stress
	•How to incorporate physical activity into everyday activities
Complications	•How diabetes affects nerves and stress level
	•How diabetes can affect teeth and gums
	•How diabetes can affect the eyes
	•Importance of foot care

### Appropriateness

Three focus groups (i.e., 1 focus group in each site) were conducted with a total of 32 participants in the intervention group at the conclusion of the last intervention class. Of the focus group participants, 74% were female and mean age was 55 years. Major themes that emerged were the relevance of the educational materials and strategies proposed to better manage blood glucose, hands-on activities, cultural aspects of the program including the stories and analogies used to convey information, and appreciation of the group format and peer educators. Intervention participants reported that the curriculum materials were easy to read and understand, and that the intervention content was more educational and interactive than other DSMES classes they had attended. They reported that the story at the beginning of each class was a useful and effective way to introduce each class topic. They liked the group format because it allowed them to share information and experiences related to medication side effects, provider interactions, and family issues relevant to diabetes self-management. As one participant explained,

“I know I’m not alone now. I’ve gotten so much out of sharing my experiences and hearing about others’ experiences with diabetes.”

When asked which activities they liked most or found most useful, participants cited the educational brochures about diabetes medications, the intervention incentives (workout towel, outrigger paddle necklace, *Dash of Aloha* cookbook), a Jeopardy-type game they played as a group to review class material, and food label reading. A young male participant said,

“The label-reading activity is great for young guys. I never learned about reading labels; I just bought whatever my mother bought or whatever was on sale.”

Participants explained that they started sharing what they learned with their families. One participant said,

“I know a lot more about diabetes now, and if my father was alive, I could have helped him more, but now I can help my daughter to prevent diabetes.”

Another participant said,

“This class really helped and now I’m teaching my grandchildren how to prevent diabetes by making healthy eating choices.”

For future interventions, participants recommended cooking classes, meal planning, and adding a diabetes support group after the intervention concluded. They also recommended using culturally based stories to promote healthy eating and physical activity in children and learning ways to discuss diabetes self-management with their employers and colleagues at work. Participants differed in their opinions about the frequency of classes, with recommendations ranging from 2 classes per week to 1 class every 2 weeks.

Overall satisfaction with Partners in Care was high, and participants thought that their involvement improved their diabetes self-management. One participant reported,

“I’ve been to a lot of other diabetes classes, and this one was the best.”

Another said,

“This information has empowered me to make better decisions about my diabetes. More knowledge, more power.”

One participant observed the positive effects of diabetes self-management in her family, stating,

“My husband is feeling much better after this class.”

Participants also reported their appreciation of the peer educators, especially their ability to explain complex concepts in plain language. Peer educators were characterized as “compassionate,” “helpful,” and “able to present information in a fun and interactive way.” They were adept at using local analogies to present information. For example, one peer educator used the analogy of a Hawaiian outrigger canoe to describe participants’ role on their diabetes care team. An outrigger canoe seats six paddlers, and all six must work together to reach their destination. The person in seat six is the steersperson who instructs the paddlers and keeps the canoe on course. In this analogy, the person with diabetes is the steersperson who guides the paddlers (i.e., everyone involved in the patient’s health and well-being, such as health care providers, relatives, and friends) to achieve his or her self-management goals.

### Peer educator implementation outcomes

#### Fidelity

The mean rating by 2 independent study staff for intervention fidelity was high (4.8 on a scale from 1 to 5), indicating that peer educators delivered at least 98% of the curriculum material as written and conducted 95% of the proposed activities. Some activities could not be delivered because of time constraints resulting from participants’ extended conversations during classes. Research staff impressions of participant understanding of class material was rated high (a mean rating of 4.7 on a scale from 1 to 5), and mean rating of satisfaction with content and activities was 3.8 on a scale from 1 to 4, indicating high satisfaction. Observers noted that participants appeared to enjoy the curriculum materials and activities as well as the group format. They frequently asked questions, engaged in discussion, and shared stories relevant to the class topic, including their own challenges with self-management. Peer educators were able to answer most questions posed by participants, and they appropriately referred participants to their health care providers in cases where they lacked the necessary knowledge or information.

#### Acceptability, adoption, appropriateness and feasibility

The main themes reported by the 3 peer educators during the focus group conducted at the end of the intervention were that the intervention materials and activities were culturally appropriate, well organized, easy to facilitate, and relevant for participants. The peer educators reported that participants enjoyed the classes, appreciated the group interaction, and enjoyed the program incentives and healthy snacks. They said that participants talked to friends and family about the program and included their families in efforts to change dietary and physical activity habits to prevent diabetes. Peer educators felt that the class time of 1–1.5 h per week was adequate, while noting that some participants’ attendance was challenged by family responsibilities, work schedules, and class frequency. Another challenge reported by peer educators was related to randomizing participants to groups in which the wait-list group would have to wait 3 months before receiving the intervention.

#### Sustainability

After the intervention concluded, one of our clinic partners adopted Partners in Care as their standard diabetes self-management education program. From 2012 to 2014, funding from the National Institutes of Minority Health and Health Disparities provided the opportunity to disseminate Partners in Care to health care and community organizations throughout Oahu. Trainings were delivered by the Partners in Care peer educators. After the training, 2 community clinics adopted Partners in Care as their standard DSMES program that is now being delivered to all patients with diabetes in those clinics. Each of the clinics have American Diabetes Association recognition as a diabetes program and are being reimbursed by Medicare for delivery of Partners in Care (2016 He Huliau Conference presentation, Honolulu, Hawaii, and personal communication with diabetes program clinic staff). In addition, one of these clinics requires medical residents to attend some of the Partners in Care classes to learn from participants their challenges with diabetes self-management. Finally, 1 community research site implemented Partners in Care a second time and offered a support group for participants to meet once per month for 6 months following the intervention.

## Discussion

Partners in Care used a community-based participatory approach to culturally adapt, implement, and evaluate an evidence-based DSMES intervention with NHPIs residing in Honolulu, Hawai’i. It is one of only a few studies that included NHPIs in a randomized controlled trial to evaluate the effectiveness of a peer-delivered DSMES intervention [28] and implementation outcomes. In 2016, we implemented another pilot of Partners in Care to evaluate the addition of social support alone offered after the intervention. As in the first pilot study, [28] participants showed significant improvement in A1C after the DSMES intervention, although there was no added benefit to A1C from subsequent social support [34]. Another implementation of Partners in Care with Latino adults demonstrated significant improvement in A1C compared to a control group and improvements in A1C were maintained at 18 months [35].

Several factors may help explain the positive results of the Partners in Care study. First, our study suggests that the use of peer educators contributed to cultural and local adaptations, retention, and active engagement of participants in the classes. Peer educators were trained to conduct interactive and constructive classes in which participants learned self-management strategies and how to put them into practice in the context of everyday life. Several studies support the use of peer educators to help patients manage their diabetes because their involvement has been shown to improve patients’ glycemic control and reduce healthcare use [36, 37]. Second, the intervention was culturally tailored with the help of local peer educators and a NHPI Steering Committee increasing the relevance of study materials. Third, the study approach incorporated and emphasized behavioral strategies and psychosocial dimensions that considered the context in which diabetes is dealt with on a daily basis [32] as opposed to traditional didactic teaching. Finally, retention was relatively high, with attrition higher among younger age participants. Family obligations and work schedules were the most frequently reported reasons for missing Partners in Care classes. Research on differing topics have also encountered higher attrition among younger participants [38–40]. Lack of sustained participation by younger individuals with type 2 diabetes is particularly concerning because the rate of decline in β-cell function appears to be more rapid [41] which contributes to premature microvascular (nephropathy, retinopathy and neuropathy) and macrovascular (cardiovascular) complications [42, 43]. Few studies have examined the pathophysiological mechanisms that contribute to the decline in β-cell function among young people [42]. Future DSMES programs should consider these demands when they schedule classes and other activities. Alternate methods of intervention delivery, such as on-line classes or technology-based interventions, may be more convenient for younger individuals who have busy work and family schedules.

Adoption and institutionalization in 3 community clinics in Hawaii has continued through 2020 and demonstrates the long-term sustainability of Partners in Care. It should be noted that these clinics are accredited diabetes programs and have the capacity to bill the Centers for Medicare and Medicaid (CMS) and individual insurance plans for the delivery of Partners in Care to patients. Achieving recognition as a diabetes program by the American Diabetes Association [44] or the Association of Diabetes Care and Education Specialists (ADCES) [45] in order to seek reimbursement for services through CMS requires several steps that may be more easily realized within healthcare institutions. For example, these recognition programs require a healthcare professional, such as a nurse educator, registered dietician, or certified diabetes educator to deliver the DSMES for reimbursement and do not allow community health workers to do so. In our study, the Federally Qualified Health clinic had a registered dietician deliver Partners in Care. This was the only study site that was an ADCES-recognized diabetes program and adopted Partners in Care as their standard DSMES after the study. Challenges to sustainability of Partners in Care and similar programs exist in settings where it may be uncommon to have mechanisms in place to request reimbursement for such services. The inability to financially support delivery of effective interventions may impede the sustainability of beneficial and effective programs in non-clinic settings.

Despite high participant satisfaction and positive results, the study has limitations. First, the high proportion of positive responses in the end-of-class surveys may reflect the voluntary nature of both the classes and the evaluation. While the surveys were anonymous, small group size and the relationship with the peer educator may have contributed to socially desirable responses. Second, fidelity during class observations may have been higher due to the study staff present at select classes. Third, some data elements, such as acceptability, adoption, and appropriateness, reported for participants may seem to overlap with those for peer educators. However, the definitions are differentiated for those elements that are the same for participants and peer educators. Fourth, the sample size in this pilot study was small and findings may not be generalizable to other NHPIs. Finally, implementation costs or cost effectiveness of the intervention was not assessed. However, delivery of interventions by peer educators in community settings typically cost less than delivery by licensed health professionals in clinical settings [46]. Although, sustained implementation of Partners in Care is being reimbursed within 3 clinical settings and may be cost effective. Without funding or mechanisms to reimburse services delivered by peer educators, sustained implementation of some programs may not be feasible in community settings. Future research should explore cost effectiveness in different settings and policies to increase reimbursement for services delivered by trained peer educators. The field of DSMES is dynamic, and approaches are constantly evolving. Further research is needed to identify effective programs to reduce diabetes-related disparities experienced by NHPI.

## Conclusions

Results from this research supports a culturally tailored, peer educator approach to DSMES among NHPIs. Understanding factors that affect implementation and adoption of self-management behaviors can inform effective approaches to improve health outcomes among NHPIs. Delivery of the Partners in Care program is feasible in health care and community settings and is a reimbursable DSMES program.

## Supplementary information

**Additional file 1.** Partners in Care participant baseline survey. The Partners in Care baseline clinical, demographic, knowledge, and behavioral survey.

## Data Availability

The datasets generated and/or analyzed during the current study are not publicly available because they contain data collected from individuals who were recruited from partnering Native Hawaiian-serving organizations. These organizations provide oversight of data collected from Native Hawaiians in this study for purposes other than this study. Data may be available from the corresponding author and the partnering Native Hawaiian organizations on reasonable request.

## Abbreviations

DSMES: Diabetes self-management education and support
NHPI: Native Hawaiians and Pacific Islanders
A1C: Hemoglobin A1C
CMS: Centers for Medicare and Medicaid (CMS)
ADCES: Association of Diabetes Care and Education Specialists (ADCES)
