# Supplementary material for: Implementation outcomes of a culturally adapted diabetes self-management education intervention for Native Hawaiians and Pacific islanders
Source: BMC Public Health. 2020 Oct 20;20:1579. doi: 10.1186/s12889-020-09690-6 (PMC7576821; doi:10.1186/s12889-020-09690-6)
Supplement: Supplementary file 1 — Additional file 1. Partners in Care participant baseline survey. The Partners in Care baseline clinical, demographic, knowledge, and behavioral survey. [file 12889_2020_9690_MOESM1_ESM.doc]

Partners in Care

Baseline Participant Survey and Clinical Form

# A. Demographics

| Participant ID: |  |  |  |  |
| --- | --- | --- | --- | --- |

| Date of Visit |  |  | – |  |  | – |  |  |  |  |  | Baseline |  | 3-Month  F/up |  | 6-Month F/up |  |
| --- | --- | --- | --- | --- | --- | --- | --- | --- | --- | --- | --- | --- | --- | --- | --- | --- | --- |

|  |  | **-** |  |  | **-** |  |  |  |  |
| --- | --- | --- | --- | --- | --- | --- | --- | --- | --- |

1. Date of birth:

2. Are you

🞏_0_ Male

🞏_1_ Female

3. What is your marital status?

🞏_0_ Never married

🞏_1_ Currently married

🞏_2_ Divorced or separated

🞏_3_ Widow/widower

4. What is the highest grade you completed in school?

| 1 | 2 | 3 | 4 | 5 | 6 | 7 | 8 | 9 | 10 | 11 | 12 | 13 | 14 | 15 | 16 | 17+ |
| --- | --- | --- | --- | --- | --- | --- | --- | --- | --- | --- | --- | --- | --- | --- | --- | --- |
| Grade School | | | | | | | | High School | | | | Vo-tech/College | | | | Post Grad |

5a. Which ethnic groups do you belong to? *(Check all that apply)*

1 Hawaiian

2 Samoan

3 Filipino

4 Marshallese

5 Micronesian *(specify)*:

6 Chinese

7 Portuguese

8 Latino/Hispanic/Chicano

9 American Indian/Alaska Native

10 Japanese

11 Korean

12 White

Other *(specify):*

5b. Circle the ethnic group you **most identify with.** (Circle only one)

6. Now I'd like to ask you a few questions about the kind of work you do. Are you:

**Interviewer: check one category**

1 Working

2 Looking for Work

3 Retired

4 Keeping house/homemaker

5 Student

6 Temporarily laid off

7 Sick or on maternity leave

8 Unpaid family worker

9 Permanently disabled

10 Other _______________________

7. Now I will read a list of ranges of yearly income. Please tell me when I get to the amount that comes closest to your total family income last year, before you paid taxes and from all sources.

**Interviewer: check one category**

1 Under $2000

2 between $2000 and $4,999

3 between $5,000 and $9,999

4 between $10,000 and $14,999

5 between $15,000 and $19,999

6 between $20,000 and $24,999

7 between $25,000 and $29,999

8 between $30,000 and $39,999

9 between $40,000 and $49,999

10 between $50,000 to $59,999

11 between $60,000 to $69,999

12 $70,000 and over

13 [Doesn't know or refuses]

14 [No response]

8. Are you participating in the PILI Ohana weight loss program?

🞏_0_ No 🞏_1_ Yes

**Clinical Data**

Time of Assessment:

Has the participant fasted for at least the past 8 hours? 🞏_0_ No 🞏_1_ Yes

1. Hemoglobin A1c value: %

2. Total Cholesterol:

LDL:

HDL:

Triglycerides:

3. Blood Pressure  Arm used for measurement

a. mm Hg Right Left

b. mm Hg

4. Height (only at baseline)

a. cm (round to the nearest 10^th^; e.g., if 69.5 cm then round up to 70 cm or if 199.4 cm then round down to 199 cm)

b. cm

5. Weight


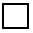
 a. **.** kg


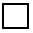
 b. **.** kg

B. General Diabetes Questions

B-1. How old were you when you were **first told that you had diabetes**?

____ Age in years

7 Don’t know/Not sure

9  Refused

B-3. Do you test your blood sugar? (check one box)

1 No _2_ Yes How many days a week do you test your blood sugar?

_____ (days / week)

**Go to question**

**B-4**

On days that you test, how many times do you test your blood sugar?

_____ (times / day)

Do you keep a record of your blood sugar test results? (check one box)

1 No _2_ Yes _3_ Only unusual values

| **B-4** | **Yes** | **No** | **Don’t Know** | **Refused** |
| --- | --- | --- | --- | --- |
| a) Are you taking diabetes pills? | 1 | 2 | 3 | 4 |
| b) Are you taking insulin? | 1 | 2 | 3 | 4 |
| c) Do you have a glucose meter used to check your blood sugar level at home? | 1 | 2 | 3 | 4 |
| d) Have you ever taken a course or class in how to manage your diabetes yourself? | 1 | 2 | 3 | 4 |

# B-5. Paying for my diabetes treatment and supplies is a problem.

1 Never

2 Sometimes/Occasionally

3 Always

4 Don't know/Not sure

5 Refused

# Diabetes Care Profile [MDRTC]

We would like to know how well you think you understand diabetes self-management activities:

| B-6 | **How do you rate your understanding of:** *(circle one answer for each question)* | **Poor** | **Somewhat** | **Good** | **Very Well** | **Excellent** |
| --- | --- | --- | --- | --- | --- | --- |
|  | a) ways to manage your diabetes | 1 | 2 | 3 | 4 | 5 |
|  | b) coping with stress | 1 | 2 | 3 | 4 | 5 |
|  | c) how food affects your blood sugar | 1 | 2 | 3 | 4 | 5 |
|  | d) the role of exercise in diabetes care | 1 | 2 | 3 | 4 | 5 |
|  | e) medications you are taking | 1 | 2 | 3 | 4 | 5 |
|  | f) how to use the results of blood sugar monitoring | 1 | 2 | 3 | 4 | 5 |
|  | g) how medicines affect blood sugar levels | 1 | 2 | 3 | 4 | 5 |
|  | h) prevention and treatment of high blood sugar | 1 | 2 | 3 | 4 | 5 |
|  | i) prevention and treatment of low blood sugar | 1 | 2 | 3 | 4 | 5 |
|  | j) prevention of long-term complications of diabetes | 1 | 2 | 3 | 4 | 5 |
|  | k) foot care | 1 | 2 | 3 | 4 | 5 |
|  | l) benefits of improving blood sugar control | 1 | 2 | 3 | 4 | 5 |

# C. Diabetes Support

# The next few questions are about the support you may receive from family and friends for your diabetes.

**C-1. My family or friends help and support me a lot to:**

| *(circle one answer for each question)* | **Strongly**  **Disagree** | **Somewhat**  **Disagree** | **Neutral** | **Somewhat**  **Agree** | **Strongly**  **Agree** | **Does**  **Not**  **Apply** |
| --- | --- | --- | --- | --- | --- | --- |
| a) follow my meal plan. | 1 | 2 | 3 | 4 | 5 | N/A |
| b) take my medicine. | 1 | 2 | 3 | 4 | 5 | N/A |
| c) take care of my feet. | 1 | 2 | 3 | 4 | 5 | N/A |
| d) get enough physical activity. | 1 | 2 | 3 | 4 | 5 | N/A |
| e) test my sugar. | 1 | 2 | 3 | 4 | 5 | N/A |
| f) handle my feelings about diabetes. | 1 | 2 | 3 | 4 | 5 | N/A |

C-2. My family or friends:

| *(circle one answer for question)* | **Strongly**  **Disagree** | **Somewhat**  **Disagree** | **Neutral** | **Somewhat** Agree | **Strongly**  **Agree** |
| --- | --- | --- | --- | --- | --- |
| a) accept me and my diabetes. | 1 | 2 | 3 | 4 | 5 |
| b) feel uncomfortable about me because of my diabetes. | 1 | 2 | 3 | 4 | 5 |
| c) encourage or reassure me about my diabetes. | 1 | 2 | 3 | 4 | 5 |
| d) discourage or upset me about my diabetes. | 1 | 2 | 3 | 4 | 5 |
| e) listen to me when I want to talk about my diabetes. | 1 | 2 | 3 | 4 | 5 |
| f) nag me about diabetes. | 1 | 2 | 3 | 4 | 5 |

C-3. Who helps you the **most** in caring for your diabetes? (check only one box)

1 Spouse

2 Other family members

3 Friends

4 Paid helper

5 Doctor

6 Nurse

7 Case manager

8 Other health care professional

9 No one

10 Other (specify):

D. Your Diabetes Self-Care

D-1. A test for hemoglobin "A one C" measures the average level of blood sugar over the past three months. About how many times in the past 12 months has a doctor, nurse, or other health professional checked you for hemoglobin "A one C"?

__ __ Number of times

0 None

98 Never heard of hemoglobin "A one C" test (**Go to D-4** )

77 Don't know/Not sure

99 Refused

D-2. What has your hemoglobin “A one C” (lab value for overall sugar control) been in the **past six months**?

1 Less than 7

2 Between 7 and 8

3 Between 8 and 9

4 Between 9 and 10

5 Greater than 10

6 Don’t know

7 Did not have one done

D-3. Based upon that value are you in:

1 Excellent condition

2 Good condition

3 Fair Condition

4 Poor Condition

D-4. Which of the following best describes what “good control” of your diabetes means to you? (Please mark only **one** choice):

1 You have few or no symptoms.

2 You are able to manage your diabetes without interfering too much with your life.

3 You have low hemoglobin “A one C”s or glycosylated hemoglobins.

4 You have normal or near normal blood glucose levels on your home blood glucose monitor.

5 Other (please specify):___________________________________________

**Summary of Diabetes Self-Care Activities** (SDSCA) (Toobert, DJ 2000)

D-5. The questions below ask you about your diabetes self-care activities during the **past 7 days.** If you were sick during the past 7 days, please think back to the last 7 days that you were not sick.

(please check one box for each question)

**On how many of the last 7 days did you:** Not at all 2-3 days 4-6 days 7 days

a. take your recommended insulin or diabetes pills? _1_  2  3  _4_

b. take your recommended insulin **dose** or **number**

of diabetes pills? _1_  2  3  _4_

c. follow a healthful eating plan? _1_  2  3  _4_

d. test your blood sugar at least as often as your

doctor has recommended? _1_  2  3  _4_

e. exercise for at least 30 minutes? _1_  2  3  _4_

f. check your feet? _1_  2  3  _4_

g. did you smoke a cigarette? _1_  2  3  _4_

**E. Problem Areas In Diabetes**

The next questions are about problem areas in diabetes that you may experience. Please tell me which of the following diabetes issues are currently a problem for you?

(Please read choices for each question)

E-1. Not having clear and concrete goals for your diabetes care?

0 1 2 3 4

Not a Minor Moderate Somewhat Serious

problem problem problem serious problem problem

E-2. Feeling discouraged with your diabetes treatment plan?

0 1 2 3 4

Not a Minor Moderate Somewhat Serious

problem problem problem serious problem problem

E-3. Feeling scared when you think about living with diabetes?

0 1 2 3 4

Not a Minor Moderate Somewhat Serious

problem problem problem serious problem problem

E-4. Uncomfortable social situations related to your diabetes care (e.g., people telling you what to eat?

0 1 2 3 4

Not a Minor Moderate Somewhat Serious

problem problem problem serious problem problem

E-5. Feelings of deprivation regarding food and meals?

0 1 2 3 4

Not a Minor Moderate Somewhat Serious

problem problem problem serious problem problem

E-6. Feeling depressed when you think about living with diabetes?

0 1 2 3 4

Not a Minor Moderate Somewhat Serious

problem problem problem serious problem problem

E-7. Not knowing if your mood or feelings are related to your diabetes?

0 1 2 3 4

Not a Minor Moderate Somewhat Serious

problem problem problem serious problem problem

E-8. Feeling overwhelmed by your diabetes?

0 1 2 3 4

Not a Minor Moderate Somewhat Serious

problem problem problem serious problem problem

E-9. Worrying about low blood sugar reactions?

0 1 2 3 4

Not a Minor Moderate Somewhat Serious

problem problem problem serious problem problem

E-10. Feeling angry when you think about living with diabetes?

0 1 2 3 4

Not a Minor Moderate Somewhat Serious

problem problem problem serious problem problem

E-11. Feeling constantly concerned about food and eating?

0 1 2 3 4

Not a Minor Moderate Somewhat Serious

problem problem problem serious problem problem

E-12. Worrying about the future and the possibility of serious complications?

0 1 2 3 4

Not a Minor Moderate Somewhat Serious

problem problem problem serious problem problem

E-13. Feelings of guilt or anxiety when you get off track with your diabetes management?

0 1 2 3 4

Not a Minor Moderate Somewhat Serious

problem problem problem serious problem problem

E-14. Not “accepting” your diabetes?

0 1 2 3 4

Not a Minor Moderate Somewhat Serious

problem problem problem serious problem problem

E-15. Feeling unsatisfied with your diabetes physician?

0 1 2 3 4

Not a Minor Moderate Somewhat Serious

problem problem problem serious problem problem

E-16. Feeling that diabetes is taking up too much of your mental and physical energy every day?

0 1 2 3 4

Not a Minor Moderate Somewhat Serious

problem problem problem serious problem problem

E-17. Feeling alone with your diabetes?

0 1 2 3 4

Not a Minor Moderate Somewhat Serious

problem problem problem serious problem problem

E-18. Feeling that your friends and family are not supportive of your diabetes management efforts?

0 1 2 3 4

Not a Minor Moderate Somewhat Serious

problem problem problem serious problem problem

E-19. Coping with complications of diabetes?

0 1 2 3 4

Not a Minor Moderate Somewhat Serious

problem problem problem serious problem problem

E-20. Feeling “burned out” by the constant effort needed to manage diabetes?

0 1 2 3 4

Not a Minor Moderate Somewhat Serious

problem problem problem serious problem problem

##### **F. Diabetes Self-Care Attitudes**

F-1. The next statements are specifically about your views about your diabetes care. How much do you agree or disagree with the following statements? [**Please read each statement and the choices for each. Circle the number that applies**.]

|  | **Strongly Disagree** | **Disagree** | **Neutral** | **Agree** | **Strongly Agree** |
| --- | --- | --- | --- | --- | --- |
| a) I feel confident in my ability to follow a healthy diet. | **1** | **2** | **3** | **4** | **5** |
| b) I am confident that I can test my blood sugar as often as my doctor tells me. | **1** | **2** | **3** | **4** | **5** |
| c) I feel confident that I can exercise for at least 30 minutes most days of the week (4-7 days/wk). | **1** | **2** | **3** | **4** | **5** |
| d) I am confident in my ability to keep my weight under control. | **1** | **2** | **3** | **4** | **5** |
| e) I am confident that I can keep my blood sugar under control. | **1** | **2** | **3** | **4** | **5** |
| f) I am confident that I can prevent complications from diabetes, such as eye and kidney problems. | **1** | **2** | **3** | **4** | **5** |

F-2. Which of the following has been the biggest problem for you in achieving or having good control of your diabetes in the **past six months**?

**[mark only one box]**

1 You have not adequately followed treatment recommendations.

2 Your medication dosage needed to be increased.

3 You need new medications.

4 You are not willing to start insulin.

5 Your disease is hard to control.

6 Other illnesses affected your diabetes.

7 Other (please specify): _______________________________________

F-3. How satisfied have YOU been with your diabetes control in the **past six months**?

1 Not at all satisfied

2 Not very satisfied

3 Pretty satisfied

4 Very satisfied

5 Completely satisfied

**G. Health Services**

**Now I would like to talk to you about some different health services that people can get.**

G-1. How many times have you seen a dietician or nutritionist (a person who does meal planning) since being diagnosed with diabetes?

1 Never

2 One time

3 2- 5 times

4 5-10 times

5 More than 10 times

6 Don’t know/Not sure

7 Refused

G-2. During the past 12 months, did you receive a teeth cleaning or dental check up

1 Yes _2_ No _3_ Don’t Know/Not sure _4_ Refused

G-3. During the past 12 months, have you had a flu shot?

1 Yes _2_ No _3_ Don’t Know/Not sure _4_ Refused

G-4. Have you ever had a pneumonia vaccination?

1 Yes _2_ No _3_ Don’t Know/Not sure _4_ Refused

G-6. When was the last time you had an eye exam in which the pupils were dilated? This would have made you temporarily sensitive to bright light and blurred your close vision.

1 Within the past month (anytime less than 1 month ago)

2 Within the past year (1 month but less than 12 months ago)

3 Within the past 2 years (1 year but less than 2 years ago)

4 2 or more years ago

8 Never

7 Don't know/Not sure

9 Refused

H. Communication

H-1. Doctors have different styles in dealing with patients. We would like to know more about how you feel about your interactions with your primary-care doctor in the **past** **six** **months**. For each statement, please tell me whether you strongly disagree, disagree, agree, or strongly agree.

|  | **Strongly Disagree** | **Disagree** | **Neutral** | **Agree** | **Strongly Agree** |
| --- | --- | --- | --- | --- | --- |
| a) You feel understood by your doctor. | **1** | **2** | **3** | **4** | **5** |
| b) You feel a lot of trust in your doctor. | **1** | **2** | **3** | **4** | **5** |
| c) Your doctor answers your questions fully and carefully | **1** | **2** | **3** | **4** | **5** |
| d) Your doctor tries to understand how you see things before suggesting a new way to do things | **1** | **2** | **3** | **4** | **5** |
| e) You get all the support that you need from your doctor | **1** | **2** | **3** | **4** | **5** |

H-2. When you last saw your doctor, how satisfied do you think your doctor was with your diabetes control?

1 Not at all satisfied

2 Not very satisfied

3 Pretty satisfied

4 Very satisfied

5 Completely satisfied

6 Don’t Know

H-3. In the last 12 months, when your diabetes doctor sent you for a blood test, x-ray or other test, how often were the results explained to you as clearly as you needed?

1 Never

2 Almost never

3 Sometimes

4 Usually

5 Almost always

6 Always

7 I did not have any medical tests in the last 6 months

H-4. If you don't understand what the health care professional tells you…

1  You ask him or her to explain it then and there.

2  You ask a nurse in the office before you leave.

3  You don't ask for an explanation from anyone at the office.

H-5. If you didn't want to do something that the doctor advised you to do…

1  You would definitely tell him/her.

2  You would ask another doctor for a second opinion.

3  You probably would not tell the doctor or ask anyone else.

H-6. Who provides you with **MOST** of the information to manage your diabetes? Please check 1-3 choices.

1 My doctor

2 A nurse or diabetes educator

3 Diabetes classes

4 My family/friends

5 Community health worker

6 Magazines or newspapers

7 Books and pamphlets

8 The Internet

9 Television

10 A support group

H-7. From whom would you **prefer** to receive most of your information on diabetes self-care?

**[Check only one:]**

1 My doctor

2 A nurse or diabetes educator

3 Diabetes classes

4 My family/friends

5 Community Health Worker

6 Magazines or newspapers

7 Books and pamphlets

8 The Internet

9 Television

10 A support group

11 No preference

**I. Food Habits and Consumption**

Now I am going to ask you some questions about foods you eat and how you prepare food.

# I-1. Diabetes Care Profile Diet Scale [MDRTC]

|  | Circle one answer. | Never | Sometimes | | | Always |
| --- | --- | --- | --- | --- | --- | --- |
| a) | How often do you follow a schedule for your meals and snacks? | 1 | 2 | 3 | 4 | 5 |
| b) | How often do you weigh or measure your food? | 1 | 2 | 3 | 4 | 5 |
| c) | How often do you (or the person who cooks your food) use the exchange lists, carbohydrate counting, or glycemic index to plan your meals? | 1 | 2 | 3 | 4 | 5 |

I-2. Are you currently on a diet or meal plan to control your diabetes?

1 Yes  2 No

I-3. How many servings of fruits and vegetables are recommended each day for a healthy diet?

1 1

2 2

3 3

4 4

5 5

I-4. Not counting juice, how often do you eat fruit? (BRFSS 317-319)

_____ Number of times **[Interviewer, enter 000 for “NEVER”]**

**Interviewer: Circle mode respondent is referring to:**

1 Per day

2 Per week

3 Per month

4 Per year

5 Don’t know/Not sure

6 Refused

I-5. How often do you eat green salad? (BRFSS 320-322)

_____ Number of times **[Interviewer, enter 000 for “NEVER”]**

**Interviewer: Circle mode respondent is referring to:**

1 Per day

2 Per week

3 Per month

4 Per year

5 Don’t know/Not sure

6 Refused

I-6. How often do you eat white rice?

_____ Number of times **[Interviewer, enter 000 for “NEVER”]**

**Interviewer: Circle mode respondent is referring to:**

1 Per day

2 Per week

3 Per month

4 Per year

5 Don’t know/Not sure

6 Refused

I-7. Not counting carrots, potatoes or salad, how many servings of vegetables do you usually eat? **A serving of vegetables at both lunch and dinner would be two servings.** (BRFSS329-331)

_____ Number of servings **[Interviewer, enter 000 for “NEVER”]**

**Interviewer: Circle mode respondent is referring to:**

1 Per day

2 Per week

3 Per month

4 Per year

5 Don’t know/Not sure

6 Refused

**I-9. The following questions are about how often you eat the following foods each week.**

| **How often do you eat…** | **0-1/wk** | **2-4/wk** | **5-7/wk** |
| --- | --- | --- | --- |
| a) high fiber whole grain bread | 1 | 2 | 3 |
| b) whole grain cereal | 1 | 2 | 3 |
| c) brown rice | 1 | 2 | 3 |
| d) beans: pinto, kidney, red, lima or navy. | 1 | 2 | 3 |
| e) multi-grain foods | 1 | 2 | 3 |
| f) legumes: split peas and lentils. | 1 | 2 | 3 |
| g) cheese, or yogurt | 1 | 2 | 3 |
| h) sweet foods such as pies, cakes, cookies, candy, sweet breads or ice cream | 1 | 2 | 3 |
| i) fried foods, fried fish, potato chips or gravy | 1 | 2 | 3 |

| I-10. **How often do you drink…** | **0-1/wk** | **2-4/wk** | **5-7/wk** |
| --- | --- | --- | --- |
| a) regular soda pop (not diet) or fruit-flavored drinks | 1 | 2 | 3 |
| b) coffee, tea or cocoa | 1 | 2 | 3 |
| c) milk, buttermilk | 1 | 2 | 3 |
| d) soymilk | 1 | 2 | 3 |

I-11. Looking at this food label, if you ate the entire bag of chips, how many total grams of carbohydrates would you eat? [DNT15]

| Nutrition Facts  Serving Size 1 oz. (28 g/About 10 chips)  Servings Per Container 2 |
| --- |
|  |
| Amount Per Serving |
| Calories 140 Calories from Fat 60 |
|  |
| % Daily Value |
| Total Fat 6 g 10% |
| Saturated Fat 0.5 g 4% |
| Cholesterol 0 mg 0% |
| Sodium 150 mg 7% |
| Total Carbohydrates 10g 6% |

1 10

2 20

3 30

4 40

5 50

6 Don't know/Not sure

7 Refused

I-12. Looking at the same food label, how many total carbohydrates are in 1 serving?

1 1

2 5

3 6

4 10

5 15

6 Don't know/Not sure

7 Refused

J. PHYSICAL ACTIVITY

We are interested in two types of physical activity: vigorous and moderate. Vigorous activities cause large increases in breathing or heart rate while moderate activities cause small increases in breathing or heart rate.

J-1. Thinking about the **moderate** physical activities you do **during your free time** in a usual week, do you do moderate activities for at least 10 minutes at a time, such as brisk walking, bicycling, vacuuming, gardening, or anything else that causes small increases in breathing or heart rate? (BRFSS 144)

1 Yes

2 No **Go to Question J-4**

3 Don’t know/Not sure **Go to Question J-4**

4 Refused **Go to Question J-4**

J-2. How many days per week do you do these moderate activities for at least 10 minutes at a time? (BRFSS 145-146)

__ __ Days per week

0 Do not exercise at least 10 minutes weekly

77 Don’t know/Not sure

99 Refused

J-3. On days when you do moderate activities for at least 10 minutes at a time, how much total time per day do you spend doing these activities? (BRFSS 147-149)

__:__ __ Hours and minutes per day

777 Don’t know/Not sure

999 Refused

J-4. Now thinking about the **vigorous** physical activities you do **during your free time** in a usual week, do you do **vigorous** activities for at least 10 minutes at a time, such as running, aerobics, heavy yard work, or anything else that causes large increases in breathing or heart rate? (BRFSS 150)

1 Yes

2 No **End of survey**

3 Don’t know/Not sure **End of survey**

4 Refused **End of survey**

J-5. How many days per week do you do these vigorous activities for at least 10 minutes at a

time? (BRFSS 151-152)

_ __ Days per week

0 Do not exercise at least 10 minutes weekly **End of survey**

77 Don’t know/Not sure

99 Refused

J-6. On days when you do vigorous activities for at least 10 minutes at a time, how much

total time per day do you spend doing these activities? (BRFSS 153-155)

__: __ __ Hours and minutes per day

777 Don’t know / Not sure

# 999 Refused
